# Subclinical muscle softening in type 2 diabetes: a shear wave elastography study linking mechanical properties to metabolic profiles

**DOI:** 10.3389/fendo.2026.1785228

**Published:** 2026-04-28

**Authors:** Wen Tang, Shuting Li, Jing Cai, Lin Chen, Tian Liu, Xiaoling Jiang, Yan Lei, Lirong Hu, Cong Chen, Lin Pu, Yuanyuan Yue

**Affiliations:** 1 Department of Ultrasound, Chengdu Integrated TCM and Western Medicine Hospital, Chengdu, China; 2 Department of Endocrinology and Metabolism, Chengdu Integrated TCM and Western Medicine Hospital, Chengdu, China; 3 Department of Health Management Medical Center, Chengdu Integrated TCM and Western Medicine Hospital, Chengdu, China; 4 Department of Ultrasound, Third People’s Hospital of Chengdu Eastern New Area, Chengdu, China

**Keywords:** diabetic myopathy, lipotoxicity, muscle quality, muscle stiffness, shear wave elastography, type 2 diabetes mellitus

## Abstract

**Background:**

Skeletal muscle is a critical target organ in type 2 diabetes mellitus (T2DM). While muscle atrophy is a well-recognized complication, qualitative tissue remodeling often precedes macroscopic volume loss. This study aimed to characterize the mechanical properties of the rectus femoris in patients with T2DM using shear wave elastography (SWE) and to investigate their associations with lipid and uric acid profiles.

**Methods:**

We conducted a cross-sectional study involving 76 patients with T2DM and 67 age- and sex-matched healthy controls. We assessed shear wave velocity (SWV), muscle thickness, and cross-sectional area of the rectus femoris bilaterally. Clinical biochemical indicators were analyzed. Physical function was evaluated using the five-repetition sit-to-stand (5STS) test and the 6-meter gait speed (6MGS) test.

**Results:**

T2DM patients exhibited significantly reduced SWV compared to controls (p < 0.001), indicating muscle softening. This mechanical alteration occurred despite preserved muscle thickness and cross-sectional area. Lower muscle stiffness was strongly correlated with elevated low-density lipoprotein cholesterol (LDL-C) and uric acid levels, but not with fasting plasma glucose. Functionally, reduced SWV was associated with prolonged 5STS duration and slower gait speed.

**Conclusion:**

T2DM impairs muscle mechanical properties prior to the onset of overt atrophy. This “quality-first” deterioration is closely linked to dyslipidemia and hyperuricemia rather than acute hyperglycemia. These findings suggest that metabolic-driven soft tissue remodeling serves as an early marker of diabetic myopathy. SWE offers a sensitive tool for detecting these subclinical changes.

## 1 Introduction

Type 2 diabetes mellitus (T2DM) represents a growing global health challenge (1). Beyond vascular complications, the disease profoundly affects skeletal muscle health (2). Muscle integrity is essential for metabolic homeostasis and locomotion. In T2DM, “diabetic myopathy” progressively compromises muscle strength and physical mobility (3–5). This decline increases the risk of falls, frailty, and loss of functional independence (6, 7). Notably, muscle quality is an independent predictor of clinical outcomes and mortality in this population, distinct from muscle mass alone (8).

Current clinical assessments primarily rely on morphological metrics, such as muscle thickness or cross-sectional area. However, quantitative atrophy is often a late manifestation of the disease. Qualitative tissue deterioration likely precedes measurable volume loss (9). The pathophysiology involves complex metabolic dysregulation, including mitochondrial dysfunction and oxidative stress (10). A central mechanism is intramuscular fat infiltration, or myosteatosis (11). Ectopic fat accumulation disrupts the mechanical architecture of the muscle and impairs force transmission (12). Detecting these microstructural changes early is crucial for preventing irreversible disability.

Shear wave elastography (SWE) is a non-invasive ultrasound technique that quantifies tissue stiffness (13, 14). It functions as a “virtual biopsy,” providing insights into tissue composition (15, 16). SWE can effectively differentiate between contractile fibers, connective tissue, and adipose infiltration (17). It has demonstrated utility in detecting muscle alterations associated with insulin resistance and fatty degeneration (18, 19). While widely applied in liver fibrosis and aging research (20, 21), the specific mechanical phenotype of early diabetic myopathy remains underdefined. Whether the muscle becomes stiffer (fibrosis) or softer (steatosis) in the initial stages of T2DM is not fully established.

## 2 Materials and methods headings

### 2.1 Study design and participants

This cross-sectional study was conducted between October 2024 and March 2025. We recruited 76 patients with T2DM from the Department of Endocrinology and Metabolism at Chengdu Integrated TCM and Western Medicine Hospital. We also recruited 67 healthy controls from the Health Management Center, matched for age and sex. Diagnosis of T2DM followed the Guidelines for the Prevention and Treatment of Diabetes Mellitus in China (2024 Edition) (22). Inclusion criteria included age between 50 and 75 years and a body mass index (BMI) of 18.5–28.0 kg/m². Exclusion criteria were severe cardiac, hepatic, or renal dysfunction, neurological disorders, recent lower limb trauma, or use of medications affecting muscle metabolism. The Research Ethics Committee of the hospital approved the study protocol.

### 2.2 Ultrasound assessment

We used an Aixplorer ultrasound system (SuperSonic Imagine, France) equipped with a 4–15 MHz linear array transducer. Participants were positioned supine with lower limbs relaxed. We identified the rectus femoris at a standardized landmark: two-fifths of the distance from the anterior superior iliac spine to the superior border of the patella (23).

**Figure 1:**
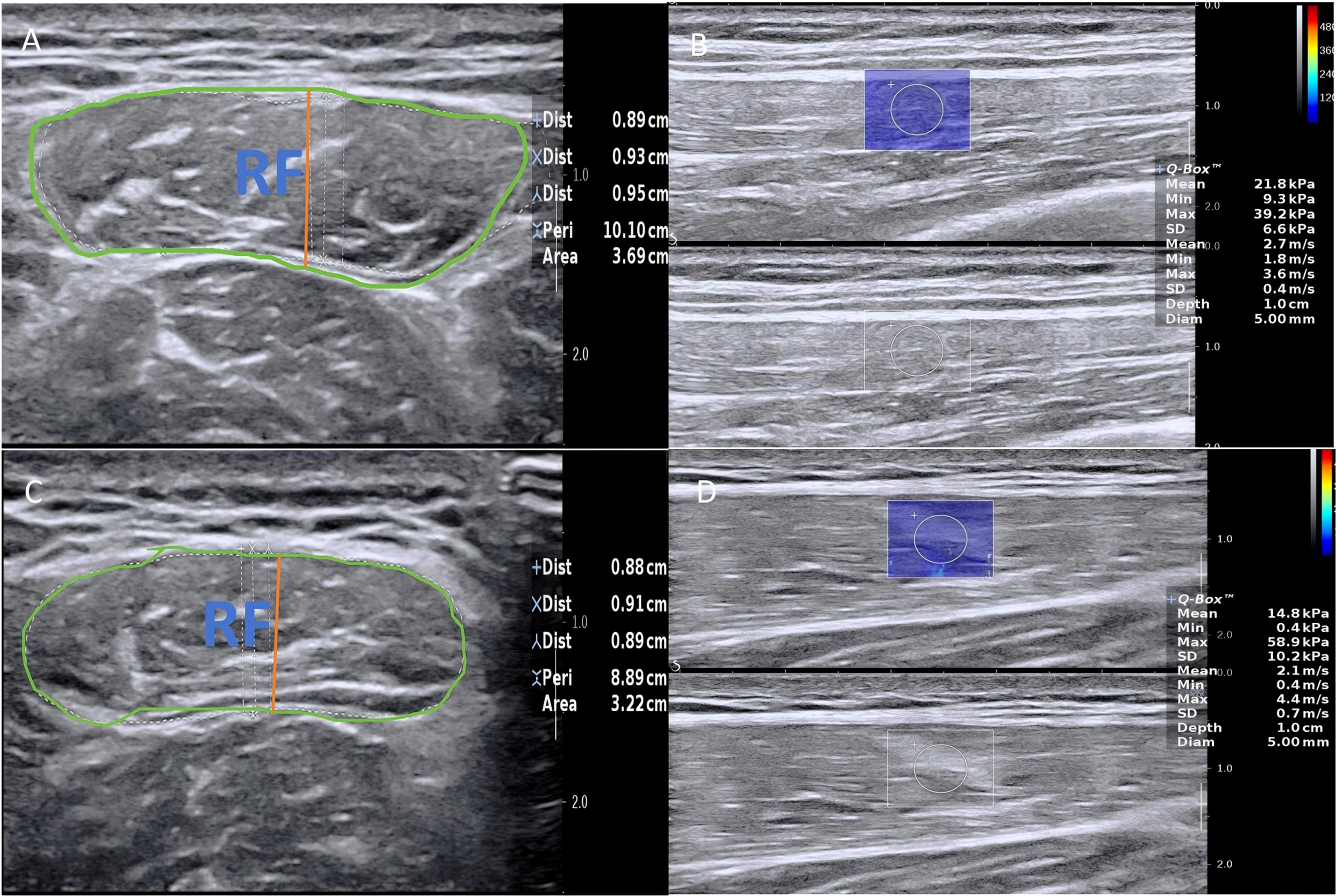
Measurement of the RF between the T2DM patients and healthy controls. Measurement of the RF between the T2DM patients and healthy controls. **(A)** Conventional two-dimensional ultrasound image of the RF in healthy controls. **(B)** Measurement of the SWV of RF in healthy controls. **(C)** Conventional two-dimensional ultrasound image of the RF in T2DM patients. **(D)** Measurement of the SWV of RF in T2DM patients.

First, B-mode ultrasound was used to measure muscle thickness and cross-sectional area (CSA). Subsequently, shear wave elastography (SWE) was performed. We placed a region of interest (ROI) within the muscle belly, carefully avoiding fascia and blood vessels (as shown in **Figure 1**). To ensure high reproducibility and minimize operator-dependent artifacts, the SWE protocol was strictly standardized. The ultrasound transducer was applied with copious coupling gel and minimal compression to avoid mechanical tissue deformation. A quantitative region of interest (ROI) with a diameter of 5 mm was carefully positioned within the thickest portion of the targeted muscle belly. We strictly ensured that the ROI was entirely confined within the muscle tissue, explicitly avoiding the surrounding echogenic fascia, aponeuroses, and macroscopic vessels to prevent boundary artifacts and anisotropic scattering. Five valid shear wave velocity (SWV) measurements were obtained per image and averaged. This procedure was repeated three times for each side, and the mean value was used for analysis.

### 2.3 Biochemical analysis

Venous blood samples were collected after an overnight fast of at least 8 hours. We analyzed fasting plasma glucose (FPG), low-density lipoprotein cholesterol (LDL-C), and uric acid (UA) levels using standard laboratory methods.

### 2.4 Functional evaluation

#### 2.4.1 Five-repetition sit-to-stand test

Participants were instructed to stand up and sit down five times as quickly as possible from a 48-cm armless chair with arms crossed. The total time was recorded (24).

#### 2.4.2 6-Meter gait speed test

Participants walked at a comfortable, usual pace along a 6-meter walkway. We calculated gait speed (m/s) based on the time taken to traverse the distance (25).

### 2.5 Statistical analysis

Data analysis was performed using SPSS software (version 27.0). Continuous variables are presented as mean ± standard deviation or median (interquartile range). Between-group comparisons were conducted using independent-sample t-tests or Mann–Whitney U tests. Reliability was assessed using intraclass correlation coefficients (ICC). Spearman correlation analysis was used to examine associations between SWV and clinical indicators. A two-sided p-value < 0.05 was considered statistically significant.

## 3 Results

### 3.1 Participant characteristics

The study included 76 T2DM patients and 67 healthy controls. There were no significant differences in age, sex, height, weight, or BMI between the groups (p > 0.05). However, T2DM patients exhibited significantly higher metabolic burden, with elevated FPG, LDL-C, and UA levels (p < 0.001). Functional performance was also impaired in the T2DM group, characterized by longer 5STS times and slower gait speeds (p < 0.001) (**Table 1**). SWE reliability was excellent, with intra- and inter-observer ICCs exceeding 0.90 (**Tables 2**, **3**).

**Table 1:** Demographic and clinical characteristics of the study population.

Basic information	Healthy controls (n=67)	T2DM patients(n=76)	P-values
Gender (Male/Fmale)	33/34	38/38	0.929
Age (Year)	63.63 ± 4.23	62.53 ± 5.83	0.204
Height (m)	1.62 ± 0.07	1.61 ± 0.07	0.748
Weight (kg)	62.94 ± 8.76	62.33 ± 8.33	0.668
BMI (kg/m 2)	23.70 ± 2.66	23.85 ± 2.52	0.724
FPG (mmol/L)	4.35 ± .65	9.40 ± 3.48	0.000
LDL-C	2.53 ± 0.64	9.40 ± 3.48	0.000
UA	198.10 ± 64.34	356.00 ± 96.31	0.000
5STS (s)	10.21 ± 1.41	12.02 ± 2.16	0.000
6MGS (m/s)	1.82 ± 0.37	1.19 ± 0.26	0.000

T2DM, type 2 diabetes mellitus; BMI, Body mass index; FPG, fasting plasma glucose; LDL-C, low-density lipoprotein cholesterol; UA, uric acid; 5STS, five-repetition sit-to-stand test; 6MGS, 6-meter gait speed.

**Table 2:** Intra-observer concordance analysis between T2DM patients and healthy controls.

Examiner	T2DM patients		Healthy controls	
	R-RF	L-RF	R-RF	L-RF
1	2.06 (1.80, 2.64)	1.96 (1.74, 2.47)	2.82 (2.45, 3.14)	2.69 (2.26, 3.06)
2	2.05 (1.82, 2.65)	1.93 (1.72, 2.49)	2.81 (2.27, 3.21)	2.36 (2.21, 3.21)
3	2.07 (1.84, 2.65)	1.93 (1.69, 2.39)	2.78 (2.25, 3.11)	2.65 (2.25, 3.02)
ICC	0.979	0.98	0.969	0.928
95% CI	0.970-.986	0.971-0.987	0.954-0.980	0.895-0.953

L, left; R, right; RF, rectus femoris; SWV, Shear wave velocity of rectus femoris.

**Table 3:** Inter-observer concordance analysis between T2DM patients and healthy controls.

Examiner	T2DM patients		Healthy controls	
	R-RF	L-RF	R-RF	L-RF
Examiner 1	2.06 (1.80, 2.64)	1.96 (1.74, 2.47)	2.82 (2.45, 3.14)	2.69 (2.26, 3.06)
Examiner 2	2.09 (1.76, 2.60)	1.98 (1.75, 2.52)	2.97 (2.36, 3.21)	2.67 (2.16, 3.15)
Examiner 3	2.13 (1.77, 2.67)	1.96 (1.75, 2.49)	2.90 (2.53, 3.21)	2.67 (2.15, 3.15)
ICC	0.987	0.989	0.979	0.94
95% CI	0.981-0.991	0.984-0.993	0.969-0.987	0.911-0.960

L, left; R, right; RF, rectus femoris.

### 3.2 Dissociation of morphology and mechanics

**Figure 2:**
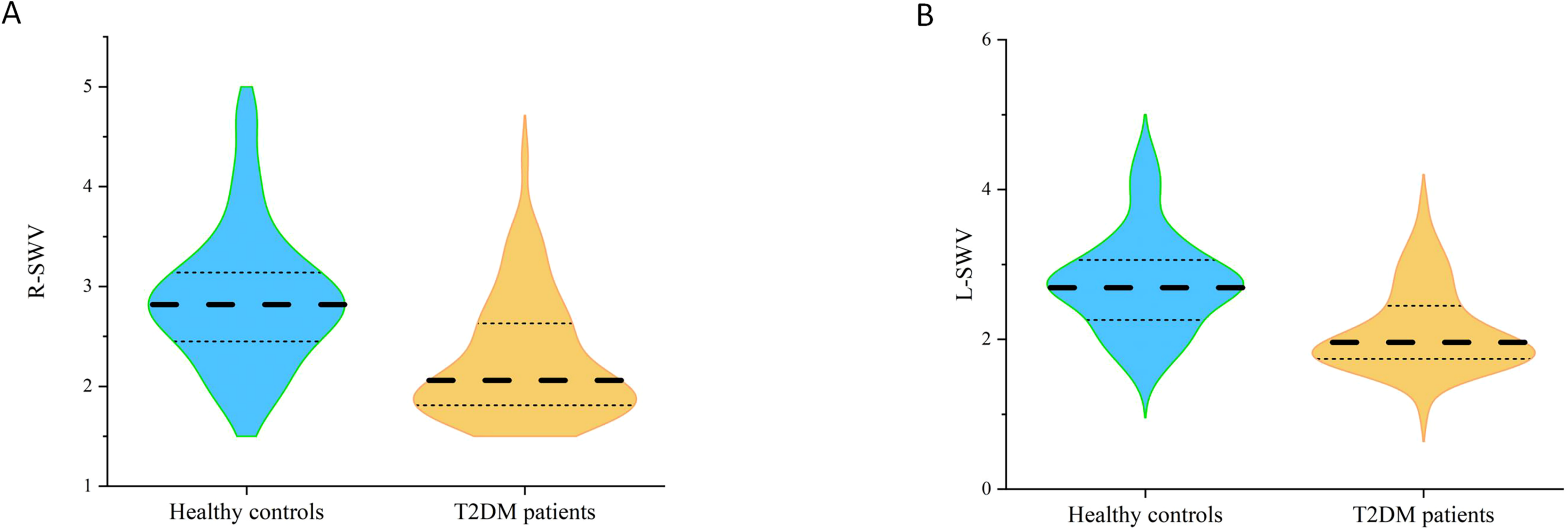
**(A)** Comparison of the SWV on the right side between T2DM patients and healthy controls. **(B)** Comparison of the SWV on the lift side between T2DM patients and healthy controls. R-SWV: Shear wave velocity of right rectus femoris. L-SWV: Shear wave velocity of left rectus femoris.

We observed a distinct dissociation between muscle structure and mechanical properties. Rectus femoris thickness and CSA did not differ significantly between T2DM patients and controls (p > 0.05). In contrast, mechanical properties were significantly altered. T2DM patients demonstrated lower SWV in the rectus femoris bilaterally (p < 0.001) (As shown in **Figure 2**; **Table 4**). This finding indicates a significant reduction in muscle stiffness compared to healthy controls.

**Table 4:** Comparison of Rectus Femoris Morphological and Mechanical Parameters between Groups.

Groups	Thickness		Cross-sectional Area		SWV	
	R	L	R	L	R	L
Healthy Controls	1.09(0.99,1.3)	1.01(0.90,1.23)	3.12(3.00,3.72)	3.01(2.82,3.49)	2.82(2.49,3.13)	2.69(2.30,3.02)
T2DM Patients	1.04(0.87,1.24)	1.02(0.92,1.09)	3.18(2.76,3.80)	3.00(2.84,3.44)	2.06(1.81,2.63)	1.96(1.74,2.45)
*P*	0.151	0.347	0.923	0.515	<0.001	<0.001

L, left; R, right; RF, rectus femoris; SWV, Shear wave velocity of rectus femoris.

### 3.3 Correlations with metabolic profiles and function

**Figure 3:**
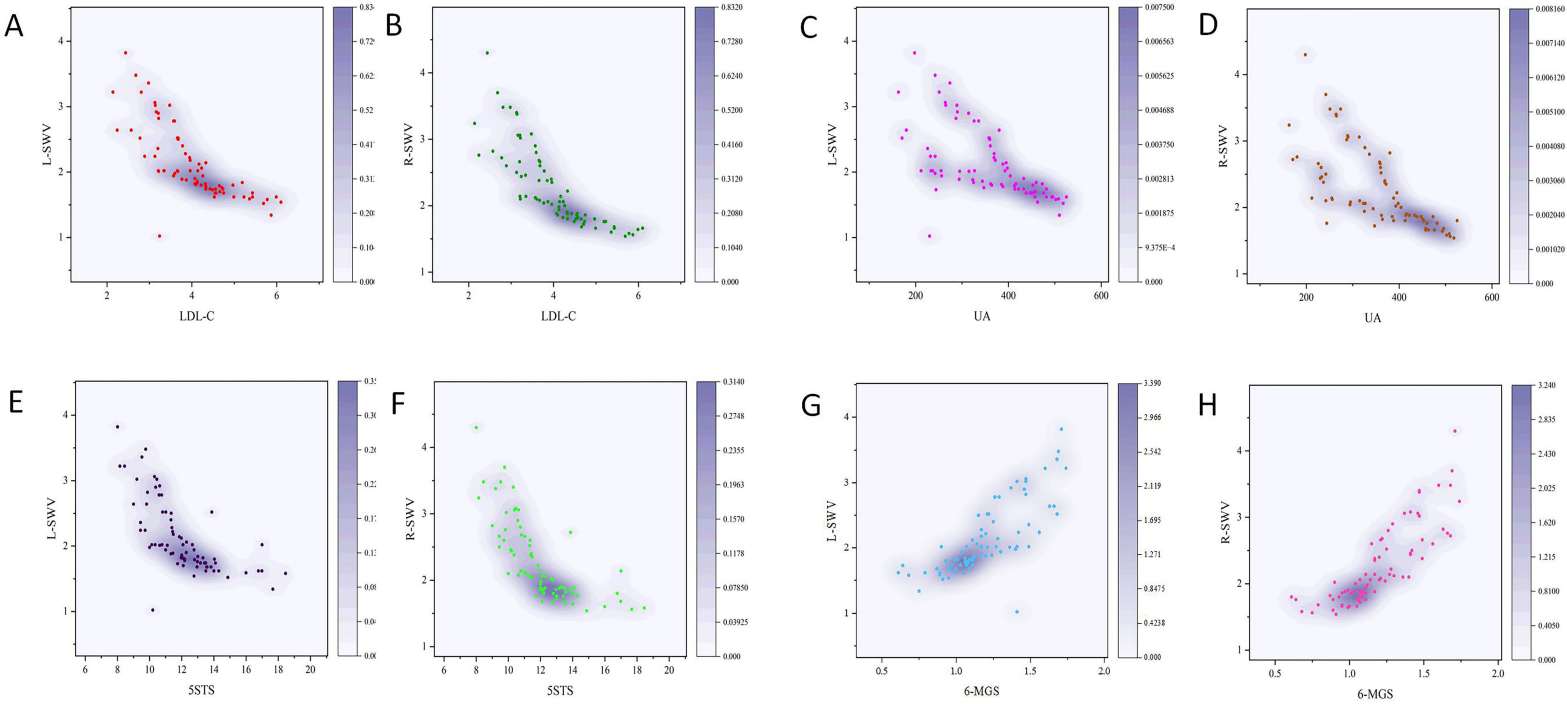
Correlation between clinical indicators and SWV on both sides in T2DM patients. **(A)** Correlation between LDL-L and L-SWV. **(B)** Correlation between LDL-L and R-SWV. **(C)** Correlation between UA and L-SWV. **(D)** Correlation between UA and R-SWV. **(E)** Correlation between 5STS and L-SWV. **(F)** Correlation between 5STS and R-SWV. **(G)** Correlation between 6MGS and L-SWV. **(H)** Correlation between 6MGS and R-SWV.L,left; R, right; RF, rectus femoris; LDL-C,low-density lipoprotein cholesterol ; UA,uric acid; 5STS, five-repetition sit-to-stand test; 6MGS, 6-meter gait speed.

In the T2DM group, reduced muscle stiffness correlated closely with adverse metabolic profiles. Lower SWV showed strong negative correlations with LDL-C (p < 0.001) and UA levels (p < 0.001). No significant correlation was observed between SWV and FPG. In terms of function, lower SWV was strongly associated with poorer physical performance. Reduced stiffness predicted longer 5STS times (p < 0.001) and slower gait speeds (p < 0.001) (As shown in **Figure 3**; **Table 5**).

**Table 5:** Correlation between US parameters and clinical Indicators in T2DM patients.

US parameters Clinical indicators	R-thickness		L-thickness		R- cross-sectional area		L- cross-sectional area		R-SWV		L-SWV	
	*r*	*P*	*r*	*P*	*r*	*P*	*r*	*P*	*r*	*P*	*r*	*P*
FPG	0.021	0.857	0.084	0.468	0.005	0.967	-0.063	0.588	-0.081	0.487	-0.051	0.664
LDL-C	-0.121	0.298	-0.061	0.602	-0.201	0.081	-0.041	0.727	-0.928^**^	0.000	-0.859^**^	0.000
UA	0.01	0.933	-0.012	0.919	-0.143	0.217	-0.006	0.959	-0.790^**^	0.000	-0.706^**^	0.000
5STS(s)	-0.14	0.229	-0.088	0.448	-0.234^*^	0.042	-0.125	0.283	-0.842^**^	0.000	-0.787^**^	0.000
6-MGS(m/s)	0.152	0.189	0.078	0.504	0.237^*^	0.039	0.059	0.612	0.895^**^	0.000	0.835^**^	0.000

L, left; R, right; RF, rectus femoris; SWV, Shear wave velocity of rectus femoris; FPG, fasting plasma glucose; LDL-C, low-density lipoprotein cholesterol; UA, uric acid; 5STS, five-repetition sit-to-stand test; 6MGS, 6-meter gait speed. The asterisk represents significant correlation.

## 4 Discussion

This study demonstrates that SWE is a sensitive tool for detecting subclinical diabetic myopathy. The primary finding is that mechanical deterioration occurs prior to overt muscle atrophy. T2DM patients exhibited reduced shear wave velocity (muscle softening) despite preserved muscle size. This supports the concept that “quality” declines before “quantity.” SWE thus offers a significant diagnostic advantage over conventional morphological assessment (26, 27).

The observed muscle softening suggests a specific metabolic etiology. While fibrosis typically increases tissue stiffness (as seen in liver disease) (20), our results align with the pathophysiology of myosteatosis. Adipose tissue is mechanically softer than contractile muscle fibers. In T2DM, systemic insulin resistance promotes ectopic lipid deposition within skeletal muscle (28). Elevated LDL-C and uric acid levels are key drivers of this process (29, 30). Our data confirm this relationship, showing strong correlations between reduced stiffness and high lipid/uric acid levels. These metabolites likely induce lipotoxicity and oxidative stress, leading to a “softening” of the muscle matrix (31).

This phenotype is consistent with findings in other metabolic and respiratory conditions. Patients with COPD and sarcopenia also exhibit reduced muscle stiffness associated with fat infiltration (32–34). This distinguishes the early diabetic muscle phenotype from inflammatory myopathies characterized by fibrosis. It is instructive to compare the muscle softening observed in our T2DM cohort with similar biomechanical alterations reported in chronic obstructive pulmonary disease (COPD). While both conditions exhibit macroscopic muscle softening—primarily associated with accelerated myosteatosis and secondary sarcopenia—their underlying pathophysiological drivers are distinct. In T2DM, the deterioration of muscle quality and the subsequent reduction in tissue stiffness are primarily mediated by systemic insulin resistance and the deleterious effects of local lipotoxicity. Conversely, the biomechanical and structural decline observed in COPD is largely driven by prolonged chronic hypoxia, physical inactivity (disuse atrophy), and heightened systemic inflammation. This distinction is critical, as it highlights that while shear wave elastography (SWE) captures similar biomechanical end-results (i.e., decreased stiffness) in both chronic diseases, the initial metabolic and molecular cascades precipitating these changes are fundamentally different.

Mechanical changes have direct functional consequences. Reduced stiffness was strongly associated with impaired sit-to-stand performance and gait speed. These tests reflect neuromuscular power and coordination (24, 25). The loss of intrinsic stiffness likely compromises the efficiency of force transmission. Therefore, SWV serves as a valuable functional biomarker that complements static anatomical measures (35). Importantly, SWE correlates well with MRI-derived fat content, reinforcing its validity as a surrogate for tissue composition (18).

We found no correlation between SWV and fasting plasma glucose. This suggests that acute hyperglycemia is not the primary driver of early structural remodeling. Instead, chronic dyslipidemia and hyperuricemia appear to play a more dominant role.

Limitations of this study include its cross-sectional design, which prevents causal inference. We did not directly measure isometric muscle strength. Future longitudinal studies are needed to validate the predictive value of these elastographic parameters for long-term disability. Furthermore, the generalizability of our findings is constrained by the specific demographic characteristics of the study cohort. Our participants were restricted to an age range of 50 to 75 years and a body mass index (BMI) between 18.5 and 28.0 kg/m². Consequently, these results may not fully extrapolate to younger individuals with early-onset T2DM, in whom the confounding effects of age-related muscle degeneration (sarcopenia) are less prominent. Finally, by excluding patients with severe obesity (BMI > 28.0 kg/m²), our study does not account for the distinct biomechanical impacts of profound adiposity. Severe obesity is typically associated with a higher degree of macroscopic intramuscular fat infiltration (myosteatosis) and altered mechanical loading, which could independently influence tissue stiffness and shear wave elastography (SWE) measurements. Future studies encompassing broader age ranges and varying degrees of adiposity are warranted to validate these findings across a more diverse diabetic population.

## 5 Conclusion

T2DM is associated with reduced rectus femoris stiffness that occurs independently of muscle atrophy. This mechanical softening is closely linked to lipid and uric acid profiles, implicating lipotoxicity in the early pathogenesis of diabetic myopathy. SWE provides a non-invasive method to detect these early qualitative changes. Identifying subclinical muscle deterioration allows for timely metabolic and rehabilitative interventions.

## Data Availability

The original contributions presented in the study are included in the article/supplementary material. Further inquiries can be directed to the corresponding author.
